# Association of Blood Heavy Metal Concentrations with Angiographic Coronary Artery Disease in a Consecutive Single-Center Cardiac Catheterization Cohort

**DOI:** 10.1007/s12012-026-10214-8

**Published:** 2026-10-27

**Authors:** Jeffrey D. Taylor, Pal Shah, Aaron Specht, Maitri Shah, Melissa Duckett, Christine Locquiao, Ken Lizama, Edward Salem, Hannah McKown, Peter Glynn, Mladen I. Vidovich

**Affiliations:** 1 Department of Cardiology, University of Illinois Chicago, Chicago, IL USA; 2 Department of Medicine, University of Illinois Chicago, Chicago, IL USA; 3 School of Health Sciences, Purdue University, Lafayette, IN USA

**Keywords:** Coronary artery disease, Heavy metals, Environmental exposure, Coronary angiography, Lead, Mercury, Cardiovascular risk factors, Social vulnerability index

## Abstract

Environmental heavy metal exposure is increasingly recognized as a potentially important but underappreciated contributor to cardiovascular disease, yet its relationship to directly observed coronary artery disease (CAD) remains poorly defined. In this single-center cohort of 52 consecutive adults undergoing cardiac catheterization at an urban Chicago tertiary care center, we paired elemental exposure profiles with direct angiographic assessment in a racially diverse, socioeconomically vulnerable population. While no individual metal independently explained obstructive CAD, circulating metal exposure patterns were more revealing: Bayesian Kernel Machine Regression demonstrated that increasing overall metal burden was associated with worse coronary phenotypes, suggesting that environmental exposures may act collectively rather than in isolation. Lead and mercury emerged as the strongest contributors and were enriched relative to National Health and Nutrition Examination Survey (NHANES) reference populations. Taken together, these findings offer early but meaningful evidence that environmental toxicant burden may represent an underrecognized layer of cardiovascular risk, particularly in urban communities where industrial history, aging infrastructure, and socioeconomic disadvantage intersect. This work highlights the importance of viewing cardiovascular disease not only through the lens of traditional risk factors, but also through the environments in which patients live.

**Graphical Abstract:**

**Figure:**
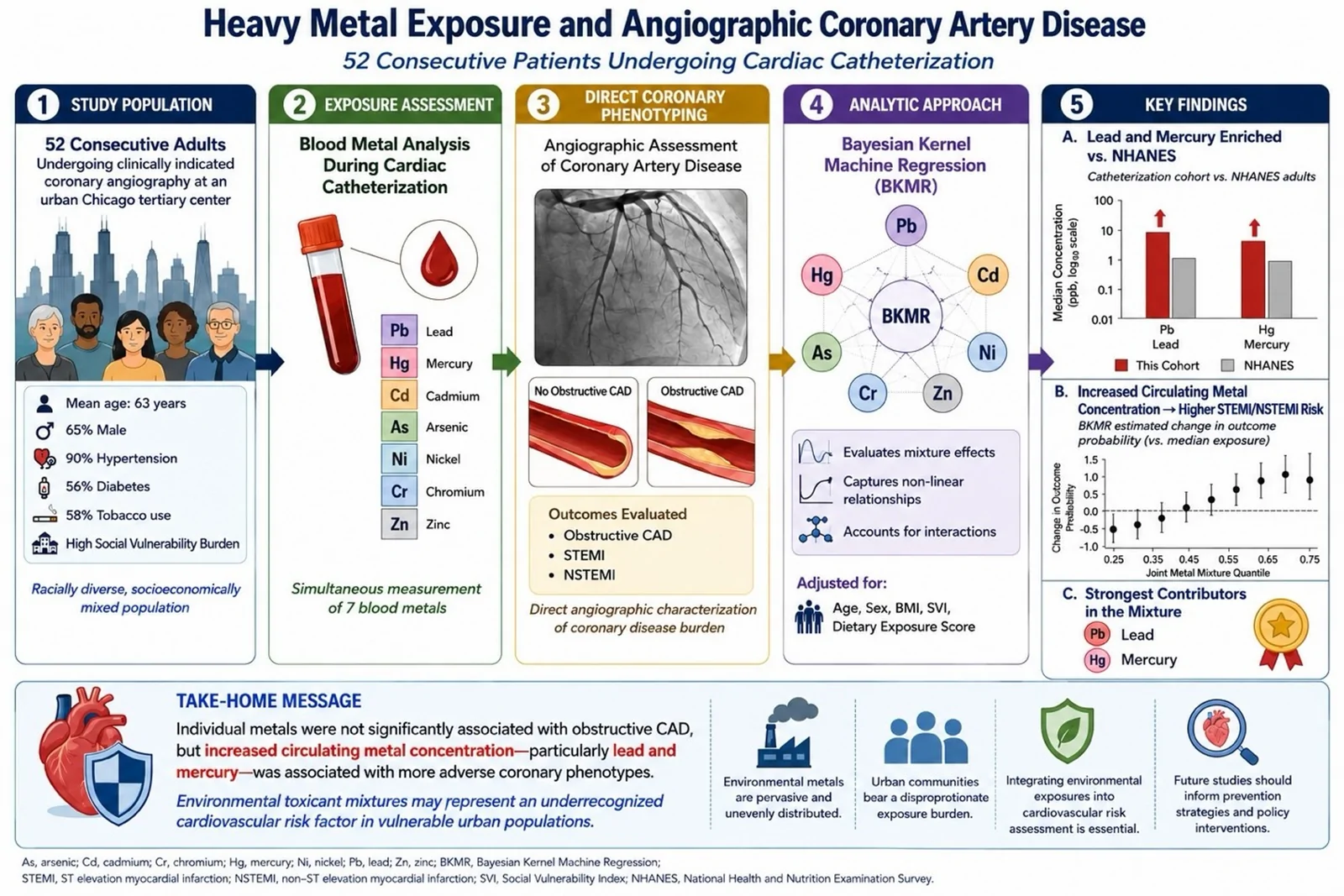

**Supplementary Information:**

The online version contains supplementary material available at https://doi.org/10.1007/s12012-026-10214-8.

## Introduction

Coronary artery disease remains the leading cause of death worldwide despite major advances in prevention and treatment. Traditional cardiovascular risk factors, including hypertension, dyslipidemia, diabetes, and tobacco use, do not fully explain the observed burden of disease, prompting growing interest in environmental determinants that may contribute to atherosclerosis through distinct mechanisms [1, 2].

Among these, heavy metals have emerged as clinically relevant yet underrecognized contributors to cardiovascular risk. Metals, including arsenic (As), cadmium (Cd), lead (Pb), mercury (Hg), chromium (Cr), nickel (Ni), and zinc (Zn), are pervasive in the environment, arising from industrial emissions, contaminated water, agricultural processes, and dietary sources. Landmark analyses from the NHANES first demonstrated associations between blood lead and cadmium levels and cardiovascular mortality, and subsequent meta-analyses have reinforced these findings, linking chronic metal exposure to coronary heart disease, stroke, and all-cause mortality [2–5]. Mechanistic studies support a causal relationship: these metals promote oxidative stress, impair endothelial function, and activate pro-inflammatory pathways central to vascular injury. Cadmium accumulates in vascular tissue and promotes plaque development; lead increases vascular stiffness; arsenic accelerates vascular aging; and mercury contributes to oxidative injury and autonomic dysregulation [2, 3, 6]. Notably, associations with cardiovascular outcomes persist even at low exposure levels, raising concern that no safe threshold may exist for certain toxic metals [2, 4, 7, 8].

Environmental metal exposure is not evenly distributed. Socioeconomically disadvantaged urban communities bear a disproportionate burden due to proximity to industrial sources, aging infrastructure, and legacy contamination from lead-based paint and plumbing. In Chicago and the broader Great Lakes region, historical steel manufacturing and industrial emissions have contributed to persistent contamination of soil, air, and water with lead, chromium, and nickel [9–12].

The present study population reflects these broader environmental and social realities. We evaluated consecutive patients undergoing cardiac catheterization at an urban tertiary care center in Chicago, serving a racially diverse, socioeconomically mixed population. Inclusion of zip-code–linked Social Vulnerability Index (SVI) data allows granular assessment of socioeconomic context in relation to metal exposure and cardiovascular risk [13].

Despite growing evidence linking heavy metals to cardiovascular disease, few studies have examined circulating metal concentrations in patients with angiographically defined CAD. Coronary angiography remains the clinical gold standard for assessing the presence and extent of obstructive disease, and establishing a link with metal exposure would provide direct evidence of an association with atherosclerotic burden. We therefore conducted a single-center observational study of consecutive catheterization patients, hypothesizing that higher circulating concentrations of selected heavy metals would be associated with both the presence and severity of CAD, independent of traditional risk factors and socioeconomic vulnerability.

## Methods

### Study Design and Population

We conducted a single-center, cross-sectional analysis of adults (≥18 years) undergoing clinically indicated cardiac catheterization at a tertiary care center. Patients with available catheter-derived elemental measurements and complete clinical data were included. Acute myocardial infarction, defined as STEMI or NSTEMI at the time of enrollment, was assessed as a clinical phenotype. The study protocol was approved by the Institutional Review Board of University of Illinois Chicago, and written informed consent was obtained from all participants prior to enrollment.

### Measurement of Metal Exposures

Elemental concentrations were measured from whole blood samples obtained during cardiac catheterization procedures. Metals were quantified using inductively coupled plasma–based methods and reported in parts per billion (ppb). Because measurements reflected localized intravascular sampling rather than peripheral venous whole blood, concentrations were interpreted as catheter-associated exposure measures at the time of cardiac catheterization.

Primary metal exposures included total arsenic (As), cadmium (Cd), lead (Pb), total mercury (Hg), chromium (Cr), nickel (Ni), and zinc (Zn). Negative values attributable to analytic variability were rounded to zero per laboratory guidance. Metal concentrations were log-transformed using log(ppb + 1) and standardized prior to modeling. The measured concentrations were interpreted as whole-blood biomonitoring measurements and were expressed in ppb, equivalent to µg/L for aqueous concentration units.

### Statistical Analysis

Baseline characteristics were summarized using means ± standard deviations for continuous variables and counts with percentages for categorical variables. Comparisons between patients with and without obstructive coronary artery disease were performed using Student’s t-tests or χ² testing as appropriate. Standardized mean differences (SMDs) were calculated to assess effect size imbalance between groups.

To evaluate the joint effects of correlated metal exposures on STEMI/NSTEMI risk, Bayesian Kernel Machine Regression (BKMR) was performed using a binomial outcome model. BKMR was selected because it accommodates correlated environmental exposures while flexibly modeling non-linear and non-additive relationships. Models incorporated age, sex, body mass index (BMI) and Social Vulnerability Index (SVI) as covariates. Markov chain Monte Carlo sampling was performed using 10,000 iterations with a 5,000-iteration burn-in period and thinning every 5 iterations. Hierarchical variable selection was used to estimate posterior inclusion probabilities. Because reference values are dependent on specimen type, age, sex, analytical method, and chemical speciation, no single physiological range was assumed for all metals. Toxic metals such as lead, cadmium, mercury, and arsenic were interpreted primarily using population biomonitoring distributions and established guidance values, whereas essential or trace elements such as copper, zinc, manganese, cobalt, and nickel were interpreted using matrix-specific reference information.

Analyses included estimation of overall mixture effects, single-metal exposure-response functions, single-metal risk summaries, and exploratory bivariate interaction surfaces. All analyses were performed in RStudio version 2024.12.1 + 563 (Posit, Boston, MA).

To contextualize cohort exposure levels, catheter-associated metal concentrations were benchmarked against NHANES 2021–2023 adult reference distributions. Survey-weighted percentiles were calculated for whole-blood lead, cadmium, and mercury concentrations using appropriate NHANES subsample weights. Blood arsenic measurements were not consistently available, therefore urinary total arsenic was used as a population-level reference comparator.

Given that cohort measurements were derived from whole-blood intravascular samples obtained during cardiac catheterization, whereas NHANES biomarkers were measured from peripheral whole blood or urine, these exposure measurements reflect distinct biological compartments and are not directly biologically equivalent. Accordingly, benchmarking analyses were intended to provide contextual population-level comparisons rather than direct exposure equivalence. ChatGPT was used to optimize visual appearance of graphical abstract and for editing.

## Results

### Baseline Characteristics

The study cohort included 52 patients, of whom 24 (46.2%) had obstructive CAD (Table 1). Mean age was 63.1 ± 10.9 years, and 65.4% of participants were male. The cohort demonstrated a substantial burden of cardiometabolic disease, including hypertension (90.4%), diabetes mellitus (55.8%), tobacco use (57.7%), and dialysis dependence (38.5%).

**Table 1:** Baseline Demographic Characteristics Stratified by Presence of Obstructive CAD

Characteristic	Overall (*N* = 52)	No Obstructive CAD (*n* = 28)	Obstructive CAD (*n* = 24)	*P* Value
Age, years	63.1 ± 10.9	61.8 ± 11.2	64.7 ± 10.5	0.34
Male sex, *n* (%)	34 (65.4)	17 (60.7)	17 (70.8)	0.57
Body mass index, kg/m²	31.8 ± 6.8	32.5 ± 7.2	31.0 ± 6.3	0.45
Social Vulnerability Index	0.61 ± 0.23	0.63 ± 0.23	0.59 ± 0.22	0.53
Creatinine, mg/dL	3.8 ± 3.6	3.4 ± 3.3	4.2 ± 3.9	0.46
Heart failure with reduced ejection fraction (HFrEF), n (%)	12 (23.1)	5 (17.9)	7 (29.2)	0.34
Hypertension, *n* (%)	47 (90.4)	25 (89.3)	22 (91.7)	1.00
Diabetes mellitus, n (%)	29 (55.8)	13 (46.4)	16 (66.7)	0.17
Tobacco use, *n* (%)	30 (57.7)	15 (53.6)	15 (62.5)	0.58
Dialysis dependence, *n* (%)	20 (38.5)	9 (32.1)	11 (45.8)	0.31
Prior stroke, *n* (%)	10 (19.2)	4 (14.3)	6 (25.0)	0.34
Cancer history, *n* (%)	8 (15.4)	3 (10.7)	5 (20.8)	0.29

Baseline demographic characteristics were generally similar between patients with and without obstructive CAD. No statistically significant differences were observed in age, sex, body mass index, social vulnerability index, creatinine, hypertension, tobacco use, dialysis dependence, or prior stroke. Patients with CAD demonstrated numerically higher rates of diabetes mellitus (66.7% vs. 46.4%) and heart failure with reduced ejection fraction (29.2% vs. 17.9%), although these differences did not reach statistical significance.

### Individual Metal Exposure Characteristics

Baseline catheter-associated metal concentrations stratified by obstructive CAD status are shown in Table 2. Across all analyzed metals, mean concentrations were numerically higher among patients with obstructive CAD compared with those without obstructive disease. However, no individual metal concentration demonstrated a statistically significant between-group difference.

**Table 2:** Baseline Blood Metal Concentrations Stratified by the Presence of Obstructive Coronary Artery Disease

Metal	Overall (*N* = 52) Mean ± SD (ppb)	No Obstructive CAD (*n* = 28)	Obstructive CAD (*n* = 24)	*P* Value
Lead (Pb)	92.4 ± 61.7	81.6 ± 54.2	105.1 ± 69.3	0.18
Cadmium (Cd)	15.2 ± 11.8	13.8 ± 10.7	16.9 ± 12.8	0.34
Mercury (Hg)	9.6 ± 8.9	8.1 ± 7.4	11.3 ± 10.1	0.21
Arsenic (As)	23.7 ± 18.5	21.5 ± 16.2	26.3 ± 20.8	0.34
Nickel (Ni)	14.9 ± 6.2	13.9 ± 5.4	16.0 ± 7.1	0.22
Copper (Cu)	27.8 ± 10.5	25.9 ± 9.2	30.1 ± 11.8	0.17
Zinc (Zn)	41.6 ± 19.3	38.7 ± 17.1	45.0 ± 21.2	0.23
Cobalt (Co)	8.1 ± 5.4	7.4 ± 4.8	9.0 ± 6.0	0.27
Manganese (Mn)	412.5 ± 685.2	368.2 ± 601.5	463.9 ± 774.1	0.41

Lead, mercury, arsenic, zinc, and copper demonstrated the largest directional differences between groups. These findings suggested a broadly elevated exposure pattern across multiple metals rather than a dominant isolated toxicant association.

### NHANES Benchmarking

**Fig. 1:**
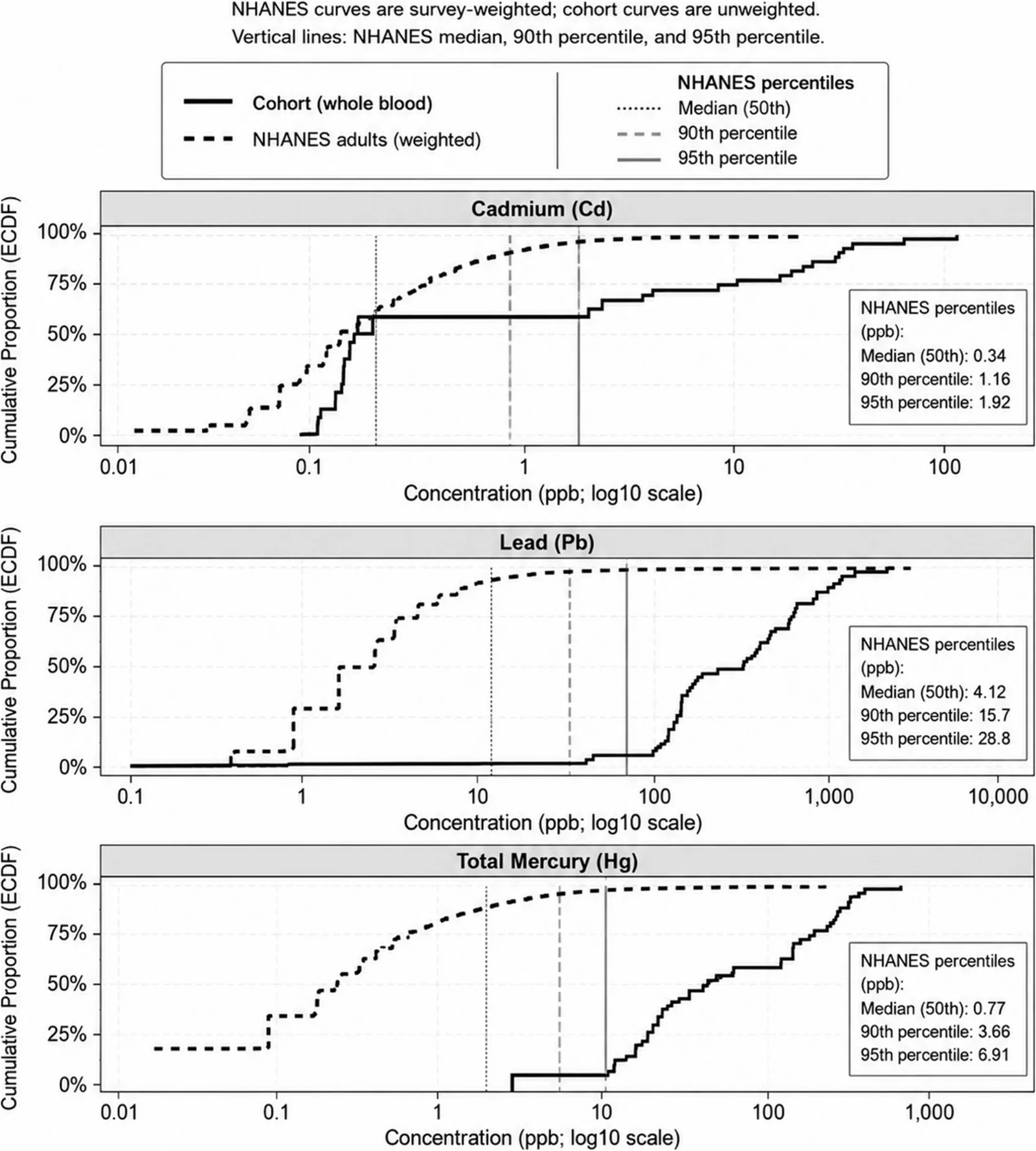
Metals Benchmarked to NHANES Adult Whole-Blood Reference Distributions. Solid black lines represent metal concentrations in the study cohort (unweighted). Dashed black lines represent survey-weighted NHANES adult whole-blood reference distributions. Vertical lines indicate NHANES median (50th), 90th percentile, and 95th percentile concentrations. Metal concentrations are shown on a log10-transformed scale (ppb)

**Fig. 2:**
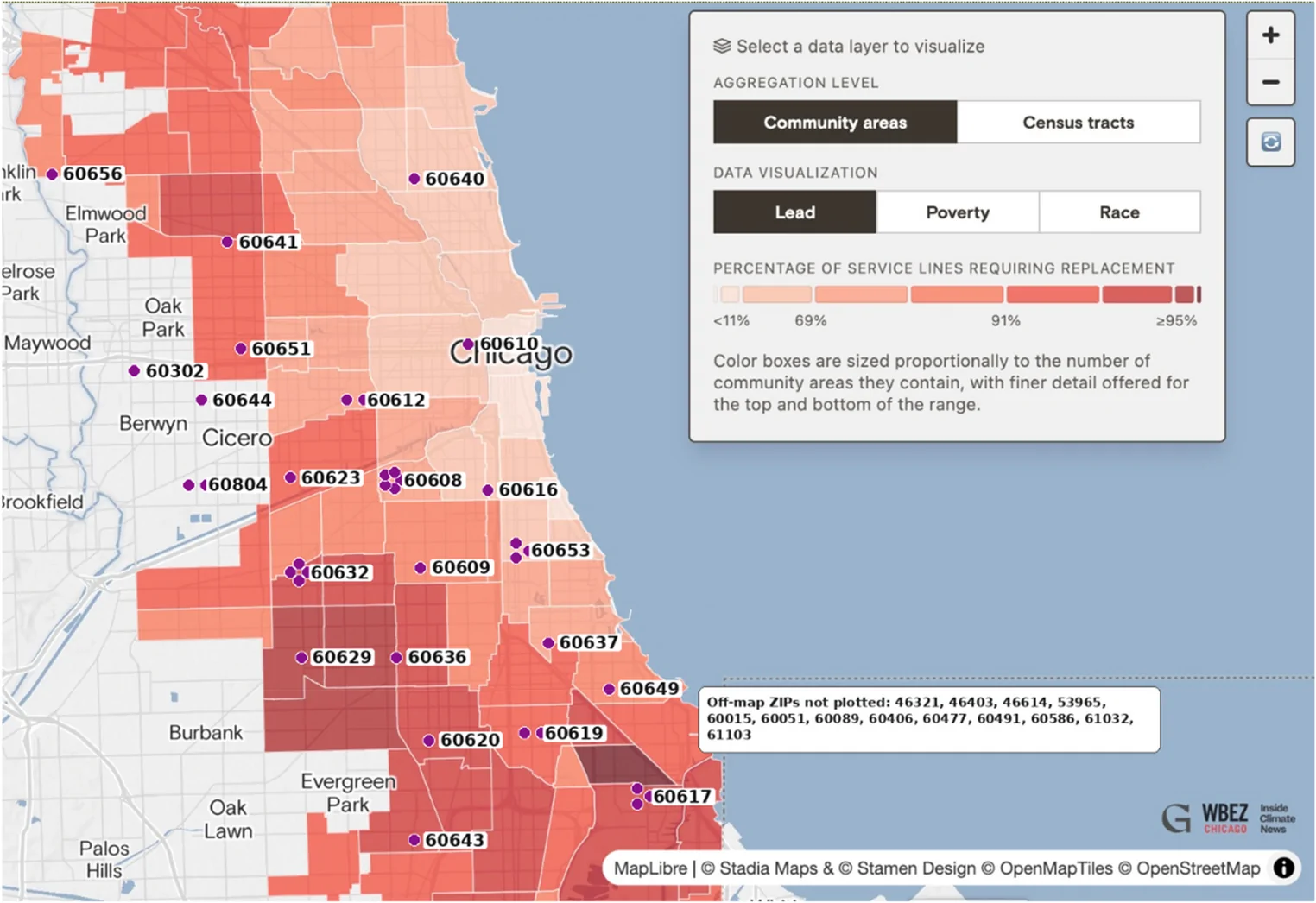
Chicago Lead Water Line Heat Map. Heat Map of Chicago water service lines in need of replacement due to lead-based pipes, patient zip codes are plotted. Data not available for zip codes outside of the city of Chicago. Data courtesy of WBEZ Chicago [14]

To contextualize observed exposure levels, whole blood metal concentrations were benchmarked against survey-weighted NHANES adult reference distributions (Fig. 1). Compared with NHANES populations, lead and mercury concentrations in the study cohort demonstrated marked rightward shifts, with a substantial proportion of cohort measurements exceeding upper NHANES percentile thresholds. The corresponding heatmap (Fig. 2) for the city of Chicago shows many participants in high-lead-exposure areas. In contrast, cadmium concentrations demonstrated greater overlap with NHANES reference distributions.

Although these measurements were obtained from different biological compartments, with intravascular whole blood sampling in the study cohort and peripheral whole-blood or urinary biomarkers in NHANES, the benchmarking analyses provide population-level context suggesting relative enrichment of several environmental metal exposures within the catheterization cohort.

### Mixture Effects of Metal Exposures

Given the correlated nature of metal exposures and the absence of strong, isolated single-metal associations, we next evaluated the effects of circulating metal exposures using BKMR.

**Fig. 3:**
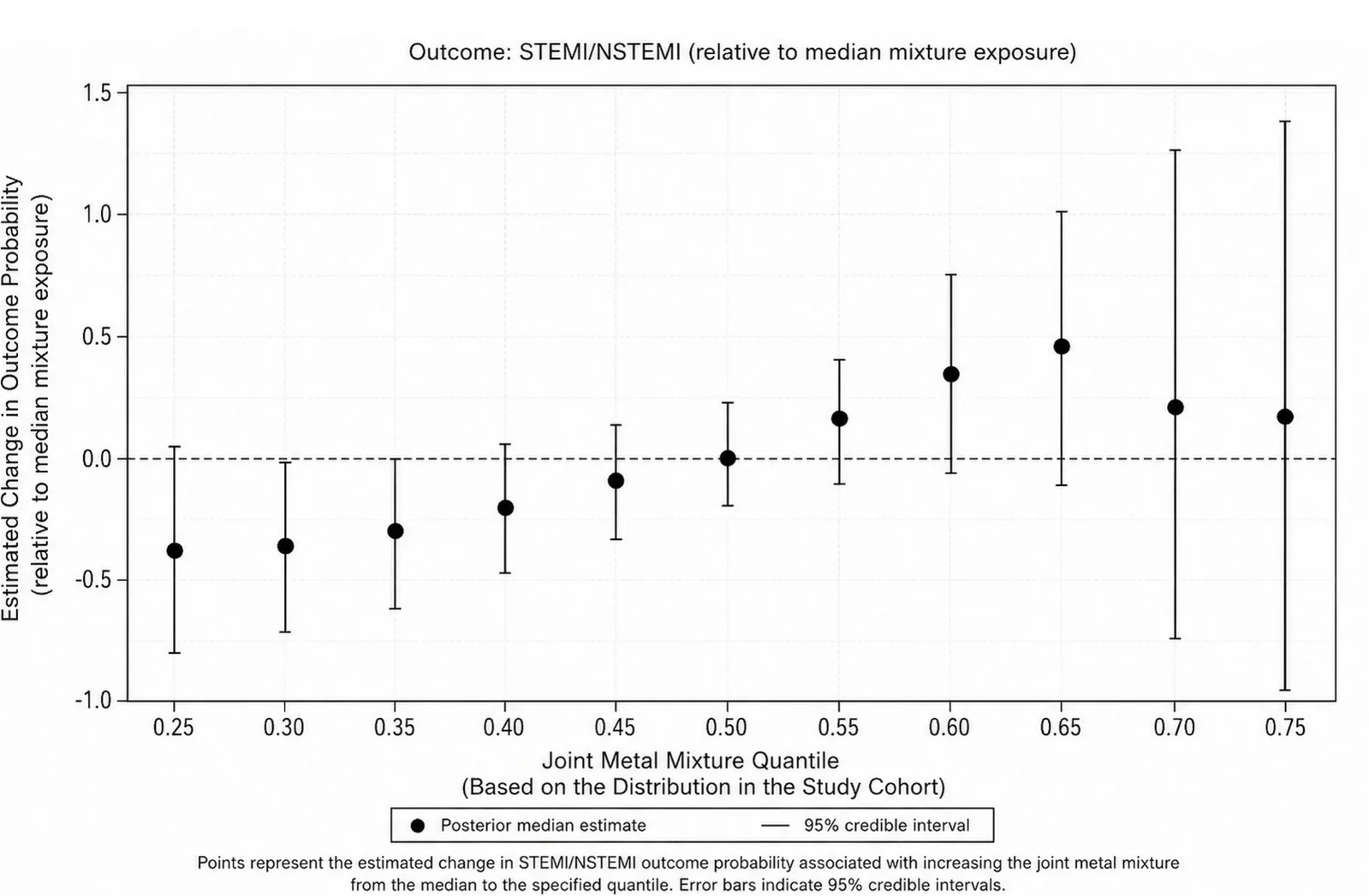
Overall Metal Mixture Effect Estimated Using Bayesian Kernel Machine Regression (BKMR)

Increasing joint metal mixture quantiles were associated with progressively higher estimated STEMI/NSTEMI outcome probability relative to the median exposure level (Fig. 3). The estimated exposure-response relationship demonstrated a monotonic upward trend across increasing mixture burden, with the greatest estimated effect observed at higher exposure quantiles.

Although credible intervals widened at the extremes of the exposure distribution, reflecting increased uncertainty at higher exposure levels, the overall directional pattern supported an exposure-response relationship across correlated metal exposures.

### Single-Metal Exposure-Response Functions

Single-metal exposure-response functions derived from BKMR models are shown in Supplemental Figure S1. Arsenic, lead, mercury, chromium, and zinc demonstrated modest positive exposure-response relationships, whereas cadmium and nickel showed comparatively flatter associations when modeled individually.

Credible intervals remained broad across all exposures, suggesting substantial uncertainty surrounding isolated single-metal effects. When all metals were simultaneously increased from lower to higher quantiles, the estimated outcome increased relative to the median exposure level, supporting a cumulative effect of the metal mixture. Confidence intervals widened at higher quantiles but remained directionally consistent with a positive exposure-response relationship.

## Discussion

In this single-center observational cohort of consecutive adults undergoing clinically indicated cardiac catheterization, several meaningful patterns emerged. Although individual heavy metal concentrations were generally higher among patients with obstructive CAD, no single metal clearly explained disease burden when examined in isolation. This finding is consistent with the recent study by Jha et al., in which individual serum concentrations of six metals were not associated with angiographic complexity as measured by the SYNTAX score in 497 patients undergoing coronary angiography [15]. Unlike Jha et al., our study classified CAD as obstructive or non-obstructive rather than using a quantitative angiographic score, which limits assessment of the full spectrum of disease severity. By contrast, when these exposures were evaluated collectively using BKMR, the combined circulating metal burden showed a more consistent relationship with adverse coronary phenotypes. This distinction is important. In real-world settings, environmental exposures rarely occur in isolation; patients are exposed to overlapping mixtures of metals through air, water, food, housing, and industrial environments. This interpretation is consistent with broader environmental cardiovascular literature, including the American Heart Association scientific statement identifying contaminant metals as important cardiovascular risk factors and contemporary reviews emphasizing combined frameworks for circulating metal exposure rather than single-agent models [1, 2].

### Looking Beyond Individual Metals: Why Mixtures May Matter More

Traditional epidemiologic studies have often focused on one metal at a time, such as lead, cadmium, or arsenic, largely because those relationships were easier to quantify. Landmark studies such as Menke et al. first demonstrated that blood lead concentrations below historically “acceptable” thresholds were still associated with increased mortality, while Chowdhury et al. later showed that arsenic, lead, cadmium, and copper were each associated with cardiovascular disease risk across multiple cohorts [4, 5]. Yet patients do not live in controlled exposure silos. Urban communities, particularly those shaped by aging infrastructure and industrial legacies, often experience simultaneous exposure to multiple pollutants. Heavy metals also share overlapping mechanisms, including oxidative stress, endothelial dysfunction, inflammation, mitochondrial injury, and vascular remodeling, which may amplify cardiovascular injury collectively even when individual exposures appear modest [1, 2, 5]. In this context, our findings support a more human and clinically realistic interpretation: the body may respond not simply to one toxic exposure, but to the biological burden of many.

### Lead and Mercury: Regionally Relevant Signals

Among the metals studied, lead and mercury appeared to contribute most strongly to mixture-associated cardiovascular risk. This observation is particularly relevant given the characteristics of our cohort: a racially diverse, largely urban Chicago population with many patients from lower socioeconomic neighborhoods. Lead exposure remains deeply tied to structural inequities, including older housing stock, legacy lead paint, water infrastructure, and contaminated urban soils [9, 10]. In Chicago specifically, contemporary studies have highlighted persistent lead exposure risks, particularly in communities shaped by historic disinvestment and older municipal systems [16]. Likewise, the Great Lakes industrial corridor has a long history of steel manufacturing, industrial emissions, and metal contamination involving lead, chromium, nickel, and other pollutants [11, 17]. Mercury enrichment may reflect additional dietary, industrial, or environmental pathways. Our NHANES benchmarking suggested that these exposures may exceed broader U.S. population norms, reinforcing the possibility that this cohort represents not only a high-risk cardiovascular population, but also one shaped by region-specific environmental burden. These findings should be interpreted cautiously, but they underscore how place, policy, and environment may shape cardiovascular biology in ways that standard risk calculators often miss.

### Environmental Exposure and Cardiovascular Equity

One of the most compelling aspects of this study is that it exists at the intersection of cardiology, public health, and environmental justice. Environmental toxicant exposure is not evenly distributed. Communities with lower socioeconomic resources are more likely to live near industrial corridors, in older housing, or in neighborhoods with persistent environmental contamination [13]. Most of our study population comes from the south and west sides of Chicago, which still have poor infrastructure to this day, as shown by the lack of lead waterlines replaced (Fig. 2). Our cohort’s relatively high SVI provides important context, suggesting that these patients may face layered cardiovascular risks extending beyond blood pressure, cholesterol, or diabetes alone. While our sample size limits definitive conclusions, the suggestion that higher social vulnerability may amplify mixture-associated cardiovascular effects is both biologically plausible and socially important. This aligns with broader literature showing that social and environmental disadvantage often cluster together, potentially accelerating cardiovascular disparities [1, 13]. For clinicians, this may represent an important reminder that cardiovascular prevention may ultimately require broader thinking, not only about medications and procedures, but about the environments in which our patients live.

### A Unique Perspective: Direct Angiographic Assessment of Coronary Disease Burden

An important strength of this study is the ability to pair circulating metal concentrations with direct angiographic characterization of coronary artery disease. Much of the existing literature examining heavy metals and cardiovascular disease has relied on population-based cohorts, cardiovascular events, mortality, or surrogate measures of atherosclerosis. Invasive coronary angiography provides patient-level assessment of epicardial coronary anatomy and allows characterization of obstructive disease, vessel involvement, and acute coronary presentation.

Recently, Jha et al. extended this literature by evaluating serum concentrations of arsenic, antimony, mercury, lead, nickel, and cadmium in 497 patients undergoing coronary angiography and examining their relationship with angiographic complexity using the SYNTAX score. Individual metal concentrations were not significantly associated with higher SYNTAX scores, although cadmium concentrations were higher among patients presenting with acute coronary syndrome compared with stable coronary disease. These findings suggest that circulating metal concentrations may not directly correspond to anatomic disease complexity and may instead demonstrate relationships with specific coronary disease phenotypes [15].

Our findings complement this work while providing a different perspective through mixture-based analysis. Similar to Jha et al., individual circulating metal concentrations in our cohort did not demonstrate consistent associations with coronary disease when considered independently. However, evaluation of the metals collectively using Bayesian kernel machine regression identified stronger associations between the circulating metal mixture and adverse coronary phenotypes, with lead and mercury contributing prominently to the observed mixture signal. Although our smaller sample size and cross-sectional design require cautious interpretation, these findings raise the possibility that evaluating correlated metal mixtures may capture relationships not apparent when individual metals are analyzed in isolation. Larger studies incorporating both quantitative angiographic scoring and mixture-based statistical approaches are needed to determine whether these associations are reproducible and whether circulating metal mixtures relate to the extent, complexity, or clinical presentation of coronary disease.

The comparison with NHANES was not intended to establish biological equivalence between catheterization-derived measurements and population biomonitoring data, but rather to contextualize circulating metal concentrations in this urban cohort relative to broader U.S. reference populations. The observed differences in lead and mercury distributions further support investigation of circulating metal profiles in populations with both high cardiovascular risk and potential environmental vulnerability.

### Limitations

Several limitations deserve acknowledgment. First and most importantly, this was a pilot study with a modest sample size. Our sample size limits power and increases uncertainty around subgroup findings. The exploratory nature of BKMR stratification means these results should be interpreted as hypothesis-generating rather than definitive. Second, observational designs preclude causal inference and residual confounding, including occupational exposures, unmeasured environmental sources, and neighborhood-level variables. Third, we acknowledge that circulating metal concentrations may be influenced by acute illness, systemic inflammation, renal function, nutritional status, redistribution between biological compartments, and renal or biliary clearance. This consideration is particularly important in our cohort because 38.5% of participants were receiving chronic hemodialysis. Finally, while the urban Chicago setting strengthens environmental relevance, it may also limit broad generalizability. At the same time, that setting is precisely what makes this study clinically meaningful: populations facing disproportionate environmental burden are often underrepresented in mechanistic cardiovascular research.

### Future Directions

This study is an early but important step toward integrating environmental exposure science into invasive cardiology. Larger multicenter cohorts are needed to validate these findings, particularly across different industrial, geographic, and socioeconomic settings. Future work should also incorporate geospatial environmental mapping, occupational histories, and prospective cardiovascular outcomes to better understand causality and long-term implications. From a mechanistic perspective, combining metal exposure profiles with coronary imaging, plaque characterization, inflammatory biomarkers, or microvascular testing could substantially deepen our understanding of how environmental toxicants shape cardiovascular disease.

## Conclusion

In this pilot cohort of consecutive patients undergoing cardiac catheterization, higher combined circulating concentrations of heavy metals, particularly lead and mercury, were associated with adverse coronary phenotypes more consistently than individual metal concentrations alone. These exploratory findings demonstrate an association between circulating metal mixtures and simultaneously assessed coronary disease but do not establish the source or duration of metal exposure, temporality, or causality. In this racially diverse, socioeconomically vulnerable urban population, our findings support further investigation into the relationship between environmental exposures and coronary artery disease. Larger, longitudinal studies incorporating repeated biomonitoring, detailed exposure assessment, and quantitative measures of coronary atherosclerotic burden are needed to validate these observations and determine their potential clinical significance. Understanding and addressing these environmental determinants may ultimately represent an important frontier in preventive and interventional cardiovascular care.

## Supplementary Information

Supplementary Material 1.

## Data Availability

All data supporting the findings of this study are available within the paper and its Supplementary Information.

## Abbreviations

CAD: Coronary artery disease
BKMR: Bayesian Kernel Machine Regression
NHANES: National Health and Nutrition Examination Survey
STEMI: ST Elevation Myocardial Infarction
NSTEMI: Non–ST elevation myocardial infarction
ppb: Parts per billion
SMDs: Standard mean differences
BMI: Body mass index
SVI: Social vulnerability index
