# Supplementary material for: Association of Blood Heavy Metal Concentrations with Angiographic Coronary Artery Disease in a Consecutive Single-Center Cardiac Catheterization Cohort
Source: Cardiovasc Toxicol. 2026 Oct 27;26(11):137. doi: 10.1007/s12012-026-10214-8 (PMC13714016; doi:10.1007/s12012-026-10214-8)
Supplement: Supplementary file 1 [file 12012_2026_10214_MOESM1_ESM.docx]

SUPPLEMENT

**Figure S1:** Single-Metal Exposure–Response Functions from Bayesian Kernel Machine Regression (BKMR) Models

*Estimated univariate exposure–response functions for individual metals included in BKMR analyses. Blue lines represent estimated changes in the outcome risk associated with increasing concentrations of each metal while holding all other metals at their median values. Gray shaded regions represent 95% credible intervals. Metal concentrations were log-transformed and standardized prior to modeling.*

**Figure S2:** Overall Metal Mixture Effect Stratified by Social Vulnerability Index (SVI)
